# Treatment of atopic dermatitis with upadacitinib: adcare single center experience

**DOI:** 10.3389/fmed.2024.1385720

**Published:** 2024-04-17

**Authors:** Daria S. Fomina, Olga A. Mukhina, Valeria I. Mikhailova, Marina S. Lebedkina, Elizaveta L. Sedova, Elena N. Bobrikova, Olga G. Elisyutina, Elena S. Fedenko, Tair T. Nurpeisov, Alexander V. Karaulov, Mar’yana A. Lysenko, Luis Felipe C. Ensina

**Affiliations:** ^1^City Clinical Hospital No. 52 of the Moscow Healthcare Department, State Budgetary Healthcare Institution, Moscow, Russia; ^2^I.M. Sechenov First Moscow State Medical University (Sechenov University), Moscow, Russia; ^3^NRC Institute of Immunology FMBA of Russia, Moscow, Russia; ^4^Peoples’ Friendship University of Russia (RUDN University), Moscow, Russia; ^5^Department of General Immunology, Asfendiyarov Kazakh National Medical University (KazNMU), Almaty, Kazakhstan; ^6^Republican Allergy Center, Research Institute of Cardiology and Internal Medicine, Almaty, Kazakhstan; ^7^Pirogov Russian National Research Medical University, Moscow, Russia; ^8^Division of Allergy, Immunology, Rheumatology, Department of Pediatrics, Federal University of São Paulo, São Paulo, Brazil

**Keywords:** upadacitinib, atopic dermatitis, drug efficacy and safety, Eczema Area and Severity Index, Janus kinase inhibitors molecules

## Abstract

**Introduction:**

The role of upadacitinib in the management of moderate to severe atopic dermatitis seems promising, but more data on its efficacy and safety are needed. This study endeavors to assess the practical impact and safety of upadacitinib in patients with moderate to severe atopic dermatitis. The study aims to evaluate the efficacy and safety of upadacitinib in the treatment of moderate to severe atopic dermatitis, focusing on analyzing patient responses to the treatment.

**Methods:**

In this study, adult patients diagnosed with moderate to severe atopic dermatitis received upadacitinib at daily doses of 15 mg or 30 mg, as prescribed by their attending physicians. The therapeutic efficacy of upadacitinib was meticulously assessed using established clinical metrics. Simultaneously, a comprehensive safety assessment was conducted through monthly monitoring, including the evaluation of potential effects of upadacitinib intake on hepatic function, lipid profile, and hematopoiesis using the pertinent laboratory tests.

**Results:**

Sixteen participants were enrolled in the study. At 1month follow-up, there was a significant reduction in the mean Eczema Area and Severity Index (EASI) score to 18.8 points, which further increased to 24 points at the 4-month mark. Additionally, 9 participants (56%) demonstrated an EASI-50 response after 1 month of treatment, with this response increasing to 9 participants (90%) after 4 months. Furthermore, enhanced therapeutic responses were observed at 4 months, with 6 patients (38%) achieving an EASI-75 response at 1month and 8 patients (80%) achieving this milestone at the 4-month follow-up. This study highlights the potential of upadacitinib as an effective treatment option for moderate to severe atopic dermatitis. While it demonstrates improved symptom management, close monitoring for potential adverse events, particularly infections and the known risks of Janus kinase inhibitors, is essential. Further research is essential to determine the long-term safety and efficacy of upadacitinib.

## Introduction

Atopic dermatitis (AD) is a common, heterogeneous, chronic, flaring, and systemic inflammatory disease characterized by eczematous skin lesions and intense pruritus and a negative impact on patients’ quality of life (QoL). It is the most common in the pediatric age group with an estimated prevalence of 15–30% (1). Nevertheless, studies have shown that up to 14% of adolescents and adults can also experience AD symptoms (2–5). However, the proportion of severe disease forms in the adult cohort is higher compared to children (6).

Adherence is essential for successful treatment. However, studies have shown that up to 33% of patients do not comply with prescribed topical therapy, and adherence drops sharply immediately following the first doctor’s visit (7). This can lead to a more severe course of the disease and an increase in skin process progression. At the same time, even with a high level of patient compliance to treatment with topical medications and a responsible attitude toward skincare, it is often not possible to achieve significant results without systemic therapy.

The therapeutic management of AD is mostly based on topical and/or systemic immunosuppressive/immunomodulant therapies and can be challenging in the long-term period, particularly for moderate-to-severe AD. Beside conventional systemic agents, such as cyclosporine, targeted therapies approved for the treatment of AD are currently available (8).

The progress of novel therapy concepts and an increased understanding of AD pathophysiology has provided the basis for new drug development for systemic therapy of moderate to severe forms of AD (3).

In the past, nonspecific immunosuppressive drugs were used, which had lack of efficacy and caused significant side effects (2, 3). Contemporary options of systemic therapy for severe atopic dermatitis have now become routine clinical practice. The pioneer in this field was dupilumab, a biological anti-IL4R monoclonal antibody. Dupilumab has demonstrated a good safety profile, although the most undesirable side effect is conjunctivitis, occurring in 4.7–28% of patients with AD. It also has good efficacy profile, effectively controlling the disease in up to 40% of patients, with time to response from 16 to 24 weeks of treatment. However, it doesn’t address all the unmet needs, i.e., it cannot be applied in patients with a tendency to inflammatory eye diseases or hypereosinophilia. In addition, the injectable form of drug administration may present additional difficulties when using dupilumab (9, 10).

Recently, the variety of targeted systemic treatment options has been further enriched with a new class of Janus Kinase inhibitors (JAKi) molecules – abrocitinib, baricitinib, and upadacitinib. The inhibition of the JAK signaling pathway is a promising therapeutic technique for reducing the activation of many pro-inflammatory mediators involved in the pathogenesis of AD. The efficacy and safety of these molecules [biologics and/or Janus kinase (JAK) inhibitors] are being evaluated in clinical trials, and several of them have already received marketing authorization. However, clinical trials are conducted in controlled situations and with selected populations, which do not necessarily reflect prescribing conditions in daily practice. Therefore, the evaluation of these molecules in real-life settings is essential for clinical practice because they assess treatment outcomes within patient populations displaying a heterogeneity of AD phenotypes (11). All molecules have received approval in clinical trials with findings indicating that upadacitinib, a selective JAK-1 inhibitor, had a slightly better safety profile at a dosage of 15 mg per day compared to Upadacitinib 30 mg per day and other classes of JAK-inhibitors in standard doses (12, 13).

Despite the fact that the results of randomized cohort studies are extremely important for evaluating the efficacy and safety of drugs, they nevertheless do not allow elucidating many issues related to the individual characteristics of patients. In these cases, the results of real clinical practice are of paramount importance.

However, the difficulty in conducting this routine clinical studylies in the current lack of consensus on what would constitute a successful treatment outcome in cases of atopic dermatitis, as the criteria used to assess response to therapy can vary significantly. These criteria include endpoints and duration of treatment.

**Objective:** To perform a step-by-step assessment of the efficacy and safety of upadacitinib for the treatment of moderate to severe atopic dermatitis in a real-world setting, analyzing patient responses to treatment.

## Materials and methods

A cohort study was conducted in two ADCARE (Atopic Dermatitis Center of Reference and Excellence) centers. The study was approved by the Ethics Committee and all participating patients provided informed consent.

A total of 19 patients (11 female) with moderate to severe AD (4 moderate AD, 15 severe AD) were candidates for targeted therapy with upadacitinib (Supplementary Diagram 1). The clinical and demographic characteristics of the cohort are presented in Table 1. Following the full examination, 3 patients withdrew their informed consent and refused to receive systemic targeted therapy. As a result, 16 patients were initiated with upadacitinib 15 mg/day from January 2022 to July 2022 inclusive. The planned dynamic monitoring period was 4 months, with 5 control visits, including the initial one. At the time of statistical data processing, six patients had not reached 4 months of follow-up. Therefore, the analysis at the 4-month follow-up was carried out for 10 patients who had entered the study earlier than the other participants.

**TABLE 1:** Patient characteristics at baseline and previous treatment strategies.

Patient characteristics (Candidates for upadacitinib treatment) (*n* = 19)	
Male,n (%)	8 (42.1)
Female,n(%)	11 (57.9)
Age, median (min-max)	33 (19–46)
BMI, median (min-max)	22.5 (16.9–40.8)
Duration of AD, median (min-max)	33 (19–46)
Family history of atopic disease,n (%)	7 (36,8)
**Previous treatmentstrategy, n (%)**	
Topical steroids	19 (100)
Emollients	19 (100)
Dupilumab	1 (5.3)
Systemic steroids	11 (57.9)
Cyclosporin	2 (10.5)
PUVA-therapy	4 (21)
**Atopic comorbidities, n (%)**	
Combination of bronchial asthma and allergic rhinoconjunctivitis Of these, severe bronchial asthma	7 (36.8) 0
Hay fever	8 (42.1)
Food allergy	6 (31.6)
None	3 (15.8)
**Allergen sensitization**	
Dust mites	12
Epidemal	13
Pollen	14
Molds	10
Food	17
Monosensitisation Of these, epidermal Of these, dust mites	3 2 1
Polysensitisation	15
Not revealed	1
**Comorbidities, n (%)**	
Obesity	1
Iron deficiency anemia	3
Beta-thalassemia	1
Sinusitis	2
Arterial hypertension	2
Tonsillitis	1
Chronic urticaria	1
**Laboratory data**	
tIgE, IU/mL, median (min-max), [normal range]	1,959 (30–8123), [0–100]
Absolute number of eosinophils, cells per microliter, median (min-max), [normal range]	325 (0–2290), [30–300]

BMI, body mass index; tIgE, total IgE.

We sourced data from individual outpatient electronic medical records and an electronic register system of a reference center. The analysis included initial patient characteristics and observations on their dynamics, in addition to results from laboratory (complete blood count, blood chemistry test, coagulation tests, total IgE levels, specific IgE levels, Hepatitis serologic tests) and instrumental (chest X-ray) tests.

Inclusion criteria were as follows:

1. Adults (at least 18 y.o.) patients irrespective of gender.
2. The presence of AD symptoms for ≥ 3 years.
3. Moderate to severe forms of atopic dermatitis (EASI ≥ 7, SCORAD ≥ 25, IGA ≥ 3; transcripts and descriptions of the questionnaires are listed below).
4. Inadequate response, such as no reduction in inflammatory foci or itching, to topical treatment with glucocorticoids and/or calcineurin inhibitors.
5. The ability to understand and fill out questionnaires related to participation in the study.
6. The ability to visit the hospital, according to the proposed plan.

Exclusion criteria were as follows:

1. Any existing contraindications to the use of upadacitinib as outlined in the product label.
2. Any conditions which would inhibit participant’s ability to engage in the study, as determined by a healthcare professional (e.g., failure to adhere to clinic visits, complete questionnaires, contact with physician due to alcohol abuse, use of illicit drugs, mental health issues, cognitive impairment etc.).
3. Refusal to consent and participate in the study at the start of treatment.

The SCORAD questionnaire was used to assess disease severity only at the first visit to evaluate if the patient was suitable for the study. Scores between 0 and 20 on this questionnaire correspond to mild AD, between 21 and 50 − to moderate AD, and above 51 − to severe AD.

The following tools were used to assess disease severity, response to treatment and quality of life throughout the study:

*– BSA (body surface area) calculated the percentage of the affected body surface.*
*– EASI (Eczema Area and Severity Index): a moderate response to therapy was considered to be a decrease in the initial index value by 50% to 75%, while an optimal response was defined as a decrease of the initial EASI by 75% to 100%. The minimum clinically significant difference or mean difference in change scores on the index was 6.6 points, with an EASI-50 defined as a minimal criterion for an early response to therapy according to* the National Institute for Health and Care Excellence (*NICE) recommendations.*

*EASI was evaluated at baseline, 1-month and 4-month follow-up.*

*– The POEM and DQLI questionnaires were used for subjective patient assessment.*

*The optimal response according to POEM (Patient-Oriented Eczema Measure) was considered 0–2 points, suboptimal (moderate)* − *3–7 points, 8 or more points* − *non-response to therapy regarding patient’s feelings. The DLQI (Dermatology Life Quality Index) had a score range from 0 to 30, with score bands indicating the following effects on patients’ lives: 0–1 indicating no effect; 6–10 indicating moderate effect; 11–20 indicating strong impact; 21–30 showing an extremely strong impact. A decrease in the DLQI index by 4 points or more after therapy was defined as a moderate response, while an optimal response was defined as a decrease to 0 or 1 score after treatment* (14). *The minimal difference in change scores is 4 points, according to the criteria for inflammatory skin diseases* (15, 16). *DLQI was evaluated at initiation, 1-month and 4-month follow-up.*

*– the numerical rating scale (NRS) of pruritus was assessed according to a 0 to 10-point scale.*
*– the disease impact on sleep (Insomnia NRS) was also observed on a 0–10-point scale* (17).
*POEM, NRS pruritis and insomnia were evaluated at baseline and at 1 month.*
*Before the treatment initiation, a hepatitis screening and QuantiFERON test were performed in order to prevent exacerbation of a possible persistent infection due to drug immunosuppression.*

Data were visualized using Statistics 13 software. The nonparametric Wilcoxon test for dependent samples was used to identify any significant differences; significance levels *p* < 0.05 (*), *p* < 0.01 (**) and *p* < 0.001 (***) were identified. The Spearman correlation coefficient was used to determine the relationship between the indicators.

## Results

### History of previous atopic dermatitis treatment strategies in the studies patients

Prior to upadacitinib initiation, one patient switched from therapy with dupilumab due to insufficient disease control. Additionally, two patients have been taking a standard dose of cyclosporine for 3 and 12 months but discontinued due to lack of efficacy. In four cases of the baseline cohort, PUVA-therapy (photochemotherapy with psoralen and A-wave ultraviolet radiation) was performed, but had an incomplete effect. Thirteen patients used systemic corticosteroids for relief during exacerbations and all patients used both topical glucocorticoids and concomitant moisturizers.

### Patient characteristics at baseline

The baseline characteristics of the primary cohort (19 patients) are presented in Table 1. According to the World Health Organization (WHO) gradation, all patients of the cohort belonged to the young age group with a median age of 33 years (ranging from 19 to 46). Early onset of atopic dermatitis (AD; within the first year of life) was reported in most patients, and the average duration of AD matched the age range and was 33 years (ranging from 19 to 46). Initially, peripheral blood eosinophilia (more than 300 cl/μl) was detected in 11 patients. High total immunoglobulin E levels (> 100UI) were observed in 16 patients. Atopy was observed in most patients before treatment with Upadacitinib (*n* = 18), with sensitization to at least one allergen as follows: dust mites (67%), epidermal (72%), pollen (14%), molds (10%) and food (17%). Polysensitization dominated with 15 out of 19 total cases, whereas monosensitization was determined in only three cases. One patient was not sensitized to any of the allergens tested.

A total of 19 patients (11 female, 8 male) with moderate to severe AD (4 moderate AD, 15 severe AD) were candidates for targeted therapy with upadacitinib (Supplementary Diagram 1). The clinical and demographic characteristics of the cohort are presented in the Table 1. After the full examination, 3 patients withdrew their informed consent and refused to receive systemic targeted therapy, thus 16 patients continued the trial and were initiated with upadacitinib 15 mg/day from January to July 2022 inclusive. The planned dynamic monitoring period was 4 months, with 5 control visits, including the initial one. Six patients had not reached 4 months of follow-up at the time of statistical data processing; therefore, the analysis at the 4-month follow-up was carried out for 10 patients who had entered the study earlier than the other participants.

**FIGURE 1:**
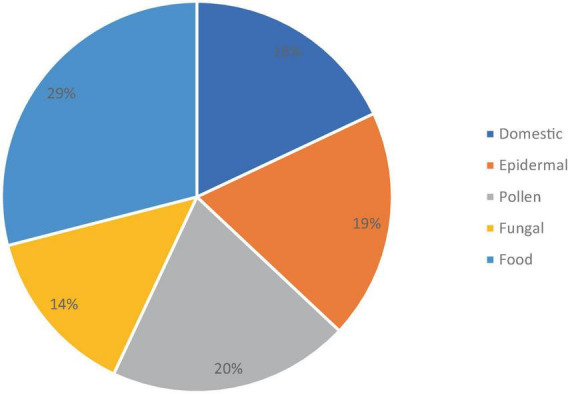
The sensitization spectrum of patients treated with upadacitinib (*n* = 16).

The sensitization spectrum of the 16 patients who have started taking upadacitinib is shown in Figure 1. Polysensitization (sensitivity to two or more groups of allergens) was observed in 12 of the 16 patients. In most cases, the patient exhibited sensitivity to multiple allergens at the same time. Monosensitization (sensitivity to only one allergen) was found in 3 patients (2 were sensitive to epidermal allergens, while one was sensitive to dust mites).

**FIGURE 2:**
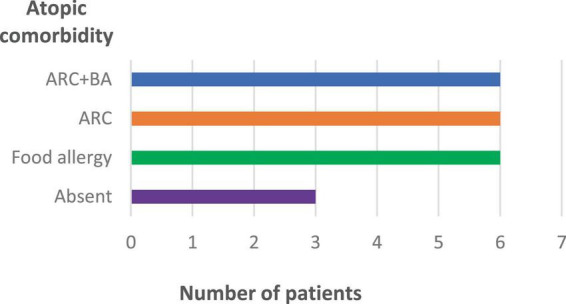
Type 2 inflammation-related comorbidities of patients treated with upadacitinib (*n* = 16). ARC, allergic rhinoconjunctivitis; BA, bronchial asthma.

Figure 2 shows the Type 2 inflammation-related comorbidities of patients treated with upadacitinib. 12 patients had rhinoconjunctivitis, 6 of whom also had bronchial asthma, and 6 patients had food allergies. Three patients had no Type 2 comorbidities.

**FIGURE 3:**
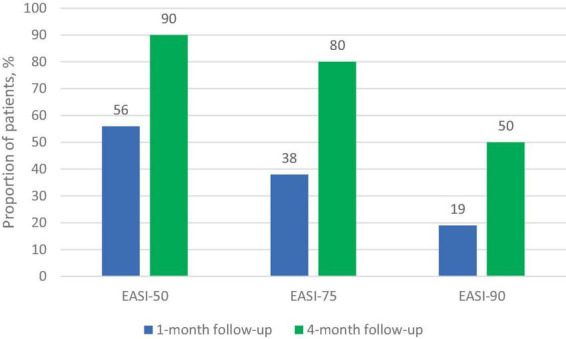
Proportion of patients achieving improvement on EASI response at 1-month and 4-month follow-up.

**FIGURE 4:**
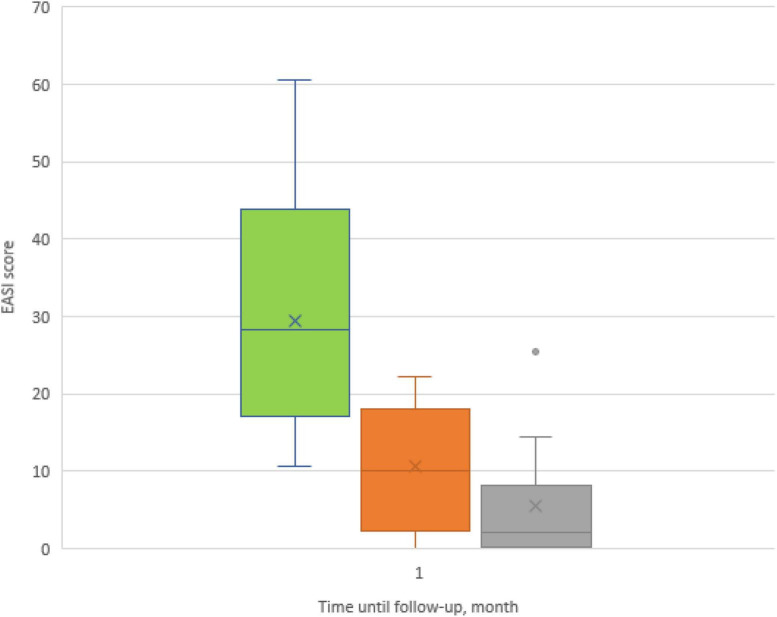
Box plots showing significant reduction in mean Eczema Area and Severity Index (EASI) score at baseline and at 1- and 4-month follow-ups.

The dynamics of the skin process under upadacitinib therapy was assessed through the EASI scores, which are represented in Figures 3, 4. At baseline, the mean EASI score was 29.5 points; a mean reduction to 18.8 points was seen at 1-month follow-up, with further gain to 24 points at 4-month follow-up (Table 2). At 1-month follow-up, 9 (56%) participants had achieved a 50% reduction in their EASI score from baseline (EASI-50), increasing to 9 (90%) at 4-month follow-up (Figure 3). Further improvement was seen toward 4 months: 6 patients (38%) achieved an EASI score decrease of 75% (EASI-75) at 1 month and 8 patients (80%) met this goal after 4 months. In addition, 50% reached the highest responses level evaluated in this study, the EASI 90 response rate, after 4 months (Figure 3). All patients were initially administered 15 mg/day of upadacitinib and 4 patients had their dose increased to 30 mg/day after 1 month due to suboptimal response.

**TABLE 2:** AD severity assessment at baseline and follow-up after 1 and 4 months on upadacitinib therapy.

Indicator	Baseline (*n* = 16)	1-month follow-up (*n* = 16)	4-month follow-up (*n* = 10)
**EASI score** Median (min-max) Mean (SD)	28.2 (10.6–60.5) 29.5(14.5)	10.1 (0–22.2) 10.7 (7.6)	2.15 (0–25.5) 5.5 (8.2)
**DLQI** Median (min-max) Mean (SD)	20.5 (4–29) 19.8 (6.7)	7.5 (0–23) 10.1 (8.0)	4 (0–20) 4.5 (5.9)
**BSA** Median (min-max)	60 (32–95)	N/A	N/A
**NRS-itch** Median (min-max) Mean (SD)	9 (5–10) 8.5 (1.6)	2 (0–8) 3.1 (2.9)	N/A
**NRS-insomnia** Median (min-max) Mean (SD)	7 (0–10) 6.5 (3.5)	1 (0–7) 2.3 (2.8)	N/A

EASI, Eczema Area and Severity Index; SCORAD, Scoring of Atopic Dermatitis; DLQI, Dermatology Life Quality Index; vIGA-AD, Validated Investigator Global Assessment for AD; BSA, body surface area; NRS, numerical rate scale; SD, standard deviation; N/A, not accessed.

In six cases, the optimal response to therapy was demonstrated 4 weeks after initiation. One patient (No. 12) only achieved a response at 3 months of therapy, but the dose wasn’t escalated due to the patient’s concerns (Table 3).

**TABLE 3:** Dynamics of skin cleansing according to the EASI index (*n* = 16).

Patients no.	EASI score at baseline	ΔEASI (%)			
		1-month follow-up	2-month follow-up	3-month follow-up	4-month follow-up
1	28.2	35	42.55	48.9	N/A
2	18	36.11	N/A	N/A	N/A
3	44.8	57.1	51.79**↑**	32.59	43.1
4	28.2	44.6	N/A	N/A	N/A
5	60.5	88.76**↑**	N/A	N/A	N/A
6	30.8	40.25**↑**	75	68.83	80.5
7	46.1	61.17**↑**	78.3	77.4	68.76
8	12.6	84.1	N/A	N/A	N/A
9	41	60**↑**	98.8	98.8	99.5
10	46.2	100	99.14	99.14	99.5
11	16.8	84.5	69.05	85.7	91.6
12	29.1	23.7	23.03	90.37	92.09
13	40,7	91,8	16,5 Skip the medication	18,04 Skip the medication	83
14	24,7	91,09	97,2	100	100
15	10,6	17,9	Skip the medication	87,7	81,13
16	14,6	42,5	N/A	N/A	N/A

N/A, not accessed. Yellow filling: achievement of moderate response; green filling: achievement of optimal response; white filling: no response, up arrow: escalation of upadacitinib dosage from 15 to 30 mg/day. The percentage difference between the EASI score at baseline and that at follow-up month is referred to as ΔEASI (ΔEASI, %). Moderate response is considered to have been achieved if the ΔEASI is 50–75%, while an optimal response is defined as a ΔEASI of 75–100%.

Three patients (No. 6, 7, 15) exhibited a wave-like pattern in their responses throughout the course of treatment, which could have been triggered by external stressors such as intense physical activity, exposure to cold air and/or allergens. Notably, patient No. 9 achieved satisfactory results with improved ΔEASI skin scores after the dose of upadacitinib was escalated to 30 mg/day during the second month of therapy. Moreover, similar benefits were observed in patients No. 6 and 7 when their daily dose of upadacitinib was escalated to 30 mg/day.

**FIGURE 5:**
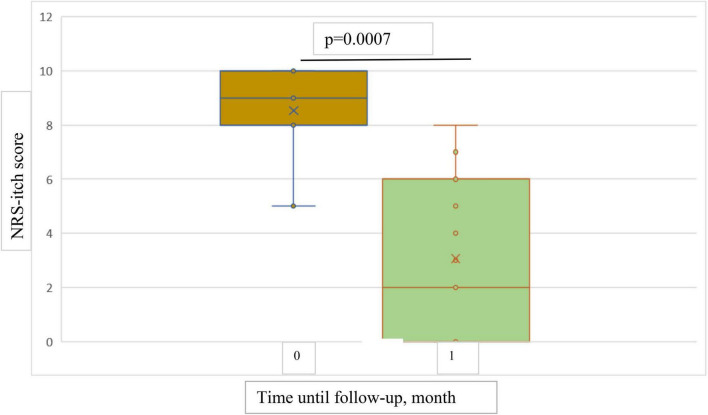
Box plots showing significant reduction in mean NRS-itch score at baseline and at 1-month follow-up.

**FIGURE 6:**
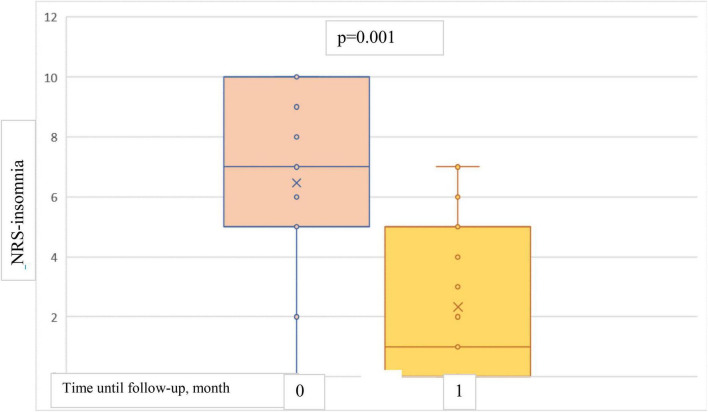
Box plots showing significant reduction in mean NRS-insomnia score at baseline and at 1-month follow-up.

The results showed a statistically significant decrease in both NRS-itch and NRS-insomnia scores from the baseline point to both 1-month and 4-months follow-up (Figures 5, 6). At the 1-month follow up, the mean percentage reduction of pruritus score from baseline was 63.5%, while the NRS insomnia score had a mean percentage reduction of 64.6% at the same time (Figure 5).

**FIGURE 7:**
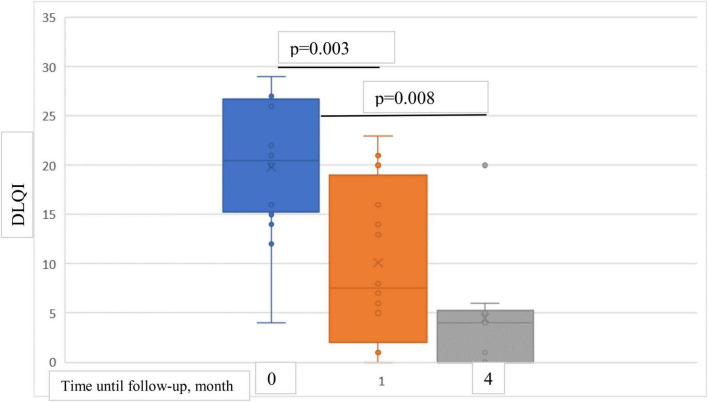
Box plots showing significant reduction in mean DLQI score at baseline and at 1- and 4-month follow-ups.

At baseline, mean DLQI score was 19.8 points, mean reduction was 9.7 points (49%) at 1-month follow-up and 14.6 points (74%) at 4-month follow-up (Figure 7).

A strong positive correlation was revealed between an increase in the quality of life and a decrease in the absolute values of EASI score (correlation coefficient = 0.966) at the 4-month follow-up.

### Treatment safety

Among 16 patients continuing treatment, 3 (18.8%) experienced acne, and 3 (18.8%) reported more frequent acute respiratory infections compared with the same period in the previous year before systemic treatment had been initiated. One patient (6.25%) noted an exacerbation of the labial form of herpetic infection; this was resolved without requiring antiviral therapy. An isolated transient elevation of ALT (100.2 u/l) was observed in one case; no other clinically significant deviations in analyses were observed.

During the study, two non-severe adverse events that led to the discontinuation of therapy were registered. A 46-year-old female reported experiencing dyspeptic phenomena (flatulence and moderate epigastric pain) during the second week of therapy. The patient chose to interrupt the treatment, and subsequently the symptoms abated. After reintroducing upadacitinib one week later, the symptoms resurfaced; thus, it was decided jointly to end the treatment.

The second case was a 28-year-old male patient who experienced acne and pyoderma periauricular skin infection after 3 weeks of treatment initiation. The symptoms resolved upon treatment discontinuation. In addition, the patient had been suffering from frequent upper respiratory viral infections up to once a month; additionally, an increase in the frequency of labial herpes infections was observed. Upon administration of oral acyclovir optimal response was seen; however, the patient decided to discontinue the treatment due to safety reasons.

## Discussion

Upadacitinib is an oral, selective and reversible small-molecule Janus kinase (JAK) inhibitor, taken once-daily. It has been specifically engineered to have greater inhibitory power against JAK1 than against JAK2, JAK3, and tyrosine kinase 2 (TYK2). JAK1 is an intracellular molecule involved in the signaling of several important cytokines that play a role in AD pathology. This medication has been approved for the treatment of moderate to severe AD in adults and adolescents over 12 years old (18).

The current study cohort showed improved signs and symptoms of AD, with a statistically significant reduction in EASI, DLQI, pruritus intensity, and sleep disorder scores from baseline to 1-month and 4-month follow-ups on upadacitinib therapy.

Of the 10 patients who reached 4 months of treatment, 8 achieved an optimal response according to the ΔEASI 75-100 criteria, 1 achieved a moderate response (ΔEASI 50-75), and 1 did not respond to therapy (ΔEASI ≤ 50).

Despite the predictable short response time to upadacitinib therapy, described in other real-world studies (19, 20), 44% of patients did not reach a ΔEASI 50 by the end of 1 month of follow-up. The scientific community has to determine an effective strategy for these cases. Options include escalating the dose of upadacitinib immediately or continuing with a standard dose for longer treatment and then assessing effectiveness criteria at week 16. However, it is certain that therapy should be continued, in the absence of any clinically significant side effects, for at least 4 months.

The timing of therapeutic response in the study by Chiricozzi et al. (8) eczema severity index (EASI) 75, EASI 90 and EASI 100 were achieved by 78.2, 47.6, and 28.2% of patients at week 16 and 87.6, 69.1, and 44.3% at week 48, respectively. The percentage of patients achieving these therapeutic goals increased until week 32, followed by a plateau.

If objective criteria of effectiveness are not met after 4 months of therapy (i.e., 1 out of 10 patients did not reach a ΔEASI-50, 2 patients did not reach a ΔEASI-75), a comprehensive assessment of the therapy’s effectiveness should be conducted by considering subjective indicators (such as POEM, DLQI, severity of itching, and sleep disorders) along with the patient. The decision to either continue or discontinue the current therapy should be jointly made by the patient and clinician; however, according to the National Institute for Health and Care Excellence (NICE) recommendations (21), failing to reach ΔEASI-50 by 4 months should most likely suggest switching/stopping the current treatment regimen.

It is interesting to note that the efficacy of upadacitinib in clinical trials was evaluated at both 16 and 52 weeks, with the primary endpoint being a ΔEASI-75, as we previously described.

The results of our study show greater effectiveness of upadacitinib therapy compared to those reported in phase III clinical trials (MEASURE-UP1, MEASURE-UP2, and AD-UP) (22–24) (Table 3). The effectiveness of the treatment was evaluated through a percentage reduction in EASI scores at 4 months, including EASI-75 and EASI-90.

A deep analysis on upadacitinib refractory patients shows that several possible factors may have contributed to this lack of response to therapy (Table 4). In addition to atopic dermatitis, female patient 3was diagnosed with a mild form of bronchial asthma and allergic rhinoconjunctivitis. Furthermore, the patient tested positive for sensitization to several groups of allergens, including dust mites and pet hairs. Upon contact with the allergens, the patient experienced shortness of breath, difficulty breathing, skin rashes, skin itching, and rhinoconjunctivitis. Additionally, patient 3was found to be sensitive to pollen (weed and tree) and various fungal allergens. Despite attempting to advise patient 3 to eliminate these factors from her environment in order to reduce inflammation and restore the epidermal barrier’s integrity, the patient refused to make any change. Furthermore, a 15-year inhalation of cigarette smoke (at 10 cigarettes per day) was noted; smoking has been shown to influence the integrity of the epidermal barrier (25) and increase pro-inflammatory immune response functions (26, 27). In addition to these factors, it is worth noting that the patient had an elevated Body Mass Index of 28.7 which may have contributed to inflammatory activity (28–30). The patient had several chronic diseases, including calculous cholecystitis, hypertension, chronic tonsillitis, dyslipidemia, and osteochondrosis with associated ischial pain syndrome. No medications to treat those comorbidities were used. It is our belief that the constant non-cutting pain syndrome has had a significant negative impact on the patient’s mental health, leading to a failure to achieve relief of subjective symptoms such as reduced itching and normalization of sleep, thus contributing to a cycle in the pathogenesis of atopic dermatitis.

**TABLE 4:** Proportion of patients who had achieved coprimary endpoints at 4 month in phase III clinical trials and current study.

ΔEASI at 4-months follow-up	MEASURE UP 1 (15 mg/day), % of patients	MEASURE UP 2 (15 mg/day), % of patients	AD UP (15 mg/day), % of patients	Current study, % of patients
EASI-75 point	69.6	60.1	64.6	80
EASI-90 point	53.1	42.4	42.8	50
EASI-100 point	16.7	14.1	–	10

There is no clear indication in the initial dose of the drug. We suggest that for adults with a long history of the disease, upadacitinib therapy should be initiated at 30 mg per day. It may take longer for a clinical effect to become apparent. Further studies are needed to confirm this hypothesis.

The results of upadacitinib clinical trials showed that most of the cohort (9), were adults aged 18-75 who had moderate to severe atopic dermatitis, with 40% of them being women (REF).

We also reviewed several case reports which highlighted the remarkable efficacy and safety of upadacitinib in patients with severe atopic dermatitis who had previously received systemic immunosuppressants or dupilumab.

The study by Dal Bello et al. (31) examined a cohort of predominantly male (80%) patients with mean age of 35 who presented with severe atopic dermatitis (median EASI score of 34) and had a history of T2 comorbidity (50% had bronchial asthma and/or allergic rhinoconjunctivitis). They had all previously been treated with various systemic therapy options (azathioprine, cyclosporine, methotrexate, UVB therapy), before being subsequently treated with dupilumab. The study showed adequate disease control on upadacitinib treatment in ten patients that failed dupilumab treatment.

The meta-analyses by Drucker et al. (32) and Sedeh et al. (33), which examined systemic therapy drugs for severe atopic dermatitis, concluded that Abrocitinib 200 mg and Upadacitinib 30 mg/day were the most acceptable treatment options. Upadacitinib 15 mg/day was also found to be equally as effective compared with Dupilumab 300 mg/2 weeks. Outcomes included change in Eczema Area and Severity Index (EASI), Patient Oriented Eczema Measure (POEM), Dermatology Life Quality Index (DLQI), and Peak Pruritus Numeric Rating Scales (PP-NRS). The drugs evaluated in these studies included Abrocitinib 100 and 200 mg, Baricitinib 2 and 4 mg, Upadacitinib 15 and 30 mg, Dupilumab 300 mg/2 weeks, and Traclokinumab 300 mg/2 weeks. In terms of safety, all treatments were generally well tolerated.

Janus Kinase (JAK) inhibitors versus Dupilumab were compared in a meta-analysis conducted by Nusbaum et al. (34), which demonstrated that the JAK inhibitor class was superior to Dupilumab regarding efficacy, without sacrificing safety. Of all JAK inhibitors evaluated, upadatinib at a dose of 30 mg showed the highest efficacy. However, the data demonstrated by Napolitano et al. (35) in a real-life dual-center experience demonstrated a statistically significant superiority of upadacitinib over dupilumab for both skin clearance and relief of pruritus, although the incidence of serious adverse events (SAEs) and adverse events (AEs) leading to discontinuation was higher in the upadacitinib-treated cohort (35).

Within the Janus Kinase Inhibitor class, an interesting meta-analysis by Zhang et al. (36) showed that upadacitinib 30 mg was superior to other oral JAK inhibitors with regard to its lack of serious adverse events. However, the cohort of patients consisted of individuals whose disease severity ranged from mild to severe.

Regarding the safety of upadacitinib, Qiu et al. (37) showed that the group receiving the escalated dose had a higher risk of acne and elevated levels of creatine phosphokinase (CPK), but no serious adverse events were detected.

According to the results of our study, as well as studies published by other authors (11, 38), severe infections and serious adverse cardiovascular events were not recorded.

As for conventional immunosuppressants, there are three main options: cyclosporine A, azathioprine, and methotrexate. According to Schram et al. (39) and Gerbens et al. (40), azathioprine is equally effective as methotrexate, as assessed by EASI, SCORAD, Skindex-17, and POEM scores. Similarly, cyclosporine A is equally effective as methotrexate, as assessed by SCORAD, EASI, and DLQI scores (41, 42). However, no comparison has been made with those using Janus kinase inhibitors.

The most frequent adverse events associated with methotrexate (MTX) are elevation of liver enzymes and gastrointestinal issues. Neurotoxicity and atrial hypertension have been reported in clinical trials involving cyclosporine. Azathioprine is associated to myelosuppression and hepatotoxicity (43).

A direct comparison was conducted in a randomized cohort trial by Blauvelt et al. (10) between upadacitinib and dupilumab (9), to ascertain if there is an equal safety profile. The results showed that, with an equal safety profile, upadacitinib was superior; at 4 month post-treatment, 71% of patients in the upadacitinib group reached EASI reference point, while only 61% of patients in the dupilumab group achieved this benchmark.

Over the past decades, our understanding of the pathophysiology of AD has grown, leading to a revolution in effective targeted therapy, such as the JAK-inhibitor upadacitinib. This is the first study to examine upadacitinib in real clinical practice with relevant issues raised concerning its dosing and response. It is important to consider both the doctor’s opinion regarding the dosing and response to therapy, as well as the patient’s point of view. Successful treatment outcomes depend on forming effective partnerships between doctors and patients that are based on communication and involve assessing both objective measures of disease activity, and subjective feelings reported by the patient.

The limitations of our study are inherent in the design of real-world registry studies. There were missing data, including clinical questionnaires and laboratory markers, for some patients. As with all registry data, there may be a bias and erroneous entery of data, limiting data quality and representativeness. Additionally, the single center data were analyzed which may be a subject to bias related to clinical practice. However, in line with the objectives of our study, at present, this represents real-life usage of this medication for AD in clinical practice.

Due to the small cohort size, the study results should be interpreted with caution, and further studies with a larger sample and longer follow-up are needed to make better recommendations for clinical use.

In conclusion, the initial real-life results with upadacitinib show promising effectiveness, even in patients with comorbidities or ones who received prior systemic treatments.

## Data availability statement

The original contributions presented in this study are included in the article/Supplementary material, further inquiries can be directed to the corresponding author.

## Ethics statement

The studies involving humans were approved by the Local Ethics Committee of City Clinical Hospital No. 52. The studies were conducted in accordance with the local legislation and institutional requirements. The participants provided their written informed consent to participate in this study.

## Author contributions

DF: Writing – original draft, Writing – review and editing, Investigation, Methodology, Project administration. OM: Data curation, Formal analysis, Investigation, Methodology, Validation, Writing – review and editing. VM: Data curation, Resources, Software, Writing – review and editing. ML: Conceptualization, Supervision, Visualization, Writing – review and editing. ES: Funding acquisition, Investigation, Validation, Writing – review and editing. EB: Data curation, Validation, Writing – review and editing. OE: Data curation, Writing – review and editing. EF: Data curation, Writing – review and editing. TN: Conceptualization, Project administration, Resources, Supervision, Writing – review and editing. AK: Conceptualization, Methodology, Supervision, Writing – review and editing. ML: Writing – original draft, Writing – review and editing. LE: Formal analysis, Project administration, Supervision, Visualization, Writing – review and editing.
