# Supplementary figures and images for: Treatment of atopic dermatitis with upadacitinib: adcare single center experience
Source: Front Med (Lausanne). 2024 Apr 17;11:1385720. doi: 10.3389/fmed.2024.1385720 (PMC11061355; doi:10.3389/fmed.2024.1385720)

# Diagram 1

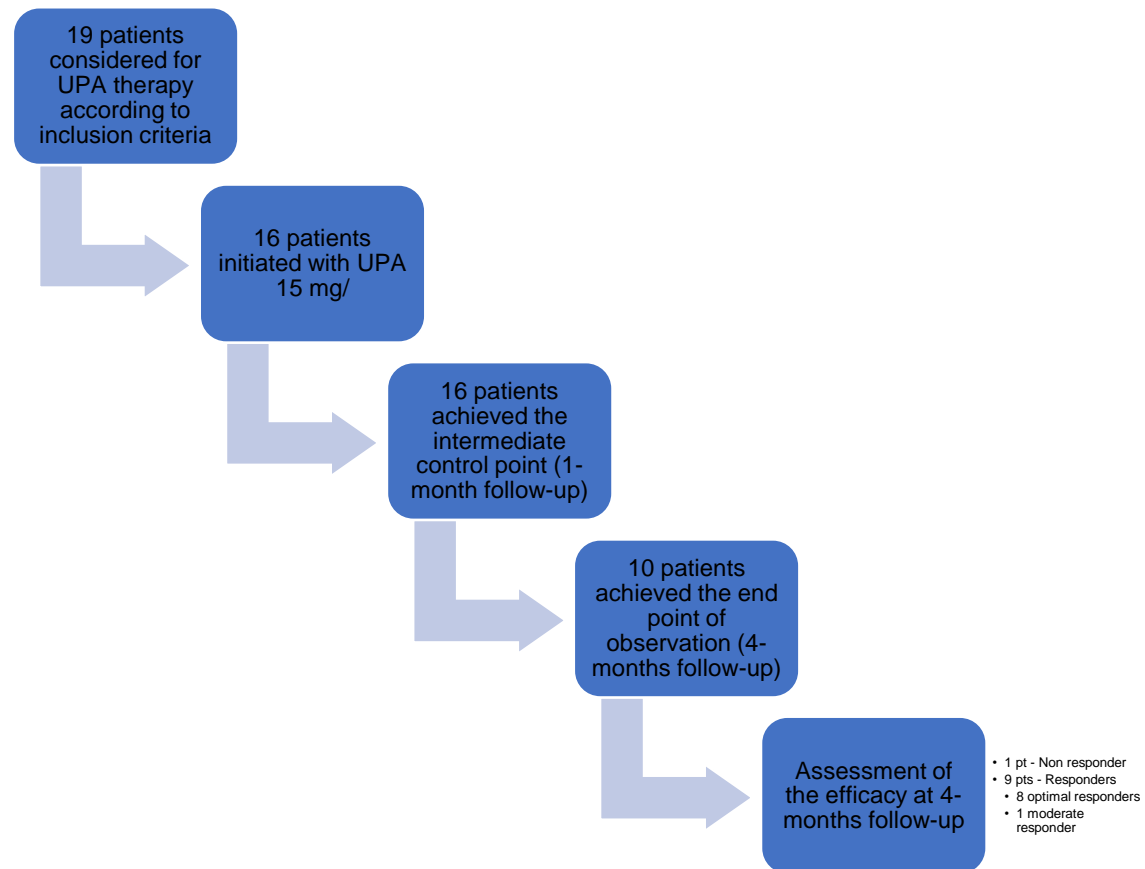

Supplement: Supplementary file 1 [file Data_Sheet_1.pdf]
